# Downmodulation of Vaccine-Induced Immunity and Protection against the Intracellular Bacterium *Francisella tularensis* by the Inhibitory Receptor Fc*γ*RIIB

**DOI:** 10.1155/2015/840842

**Published:** 2015-04-19

**Authors:** Brian J. Franz, Ying Li, Constantine Bitsaktsis, Bibiana V. Iglesias, Giang Pham, Raju Sunagar, Sudeep Kumar, Edmund J. Gosselin

**Affiliations:** ^1^Center for Immunology and Microbial Disease, Albany Medical College, Albany, NY 12208, USA; ^2^Regeneron Pharmaceuticals, 81 Columbia Turnpike, Rensselaer, NY 12144, USA; ^3^Department of Biological Sciences, Seton Hall University, South Orange, NJ 07079, USA; ^4^Regeneron Pharmaceuticals, 777 Old Saw Mill River Road, Tarrytown, NY 10591, USA; ^5^Pfizer, 610 Main Street, Cambridge, MA 02139, USA

## Abstract

Fc gamma receptor IIB (Fc*γ*RIIB) is the only Fc gamma receptor (Fc*γ*R) which negatively regulates the immune response, when engaged by antigen- (Ag-) antibody (Ab) complexes. Thus, the generation of Ag-specific IgG in response to infection or immunization has the potential to downmodulate immune protection against infection. Therefore, we sought to determine the impact of Fc*γ*RIIB on immune protection against *Francisella tularensis* (*Ft*), a Category A biothreat agent. We utilized inactivated *Ft* (i*Ft*) as an immunogen. Naïve and i*Ft*-immunized Fc*γ*RIIB knockout (KO) or wildtype (WT) mice were challenged with *Ft*-live vaccine strain (LVS). While no significant difference in survival between naïve Fc*γ*RIIB KO versus WT mice was observed, i*Ft*-immunized Fc*γ*RIIB KO mice were significantly better protected than i*Ft*-immunized WT mice. *Ft*-specific IgA in serum and bronchial alveolar lavage, as well as IFN-*γ*, IL-10, and TNF-*α* production by splenocytes harvested from i*Ft*-immunized Fc*γ*RIIB KO, were also significantly elevated. In addition, i*Ft*-immunized Fc*γ*RIIB KO mice exhibited a reduction in proinflammatory cytokine levels *in vivo* at 5 days after challenge, which correlates with increased survival following *Ft*-LVS challenge in published studies. Thus, these studies demonstrate for the first time the ability of Fc*γ*RIIB to regulate vaccine-induced IgA production and downmodulate immunity and protection. The immune mechanisms behind the above observations and their potential impact on vaccine development are discussed.

## 1. Introduction

Fc gamma receptor IIB (Fc*γ*RIIB), which is expressed on B cells, as well as other antigen (Ag) presenting cells (APC), is the only Fc gamma receptor (Fc*γ*R) known to negatively regulate the immune response and play an important role in B-cell regulation and antibody (Ab) production [1–7]. Thus, Ag-specific Ab generated in response to infection, immunization, or the administration of Fc*γ*R-targeted vaccines, when complexed with Ag, has the potential to interact with Fc*γ*RIIB and significantly downmodulate immunity and protection against infectious agents. Importantly, Fc*γ*RIIB is also expressed on dendritic cells (DCs) and macrophages and can negatively regulate cellular as well as humoral immunity [1–3, 6, 8]. Consistent with the regulation of cellular immunity, it has been demonstrated that utilizing a* Mycobacterium tuberculosis* (MTB) model in which naïve wildtype (WT) and Fc*γ*RIIB knockout (KO) mice received an aerosol challenge of MTB, Fc*γ*RIIB KO mice exhibited an enhanced cellular immune response compared to their WT counterparts, which included increased IFN-*γ* production [9]. In another study, following* Staphylococcus aureus* (SA) infection, naïve Fc*γ*RIIB KO mice were better protected against a primary intravenous infection with SA [10]. However, in contrast to the above results using naïve mice, if Fc*γ*RIIB KO mice were first immunized with a pneumococcal vaccine and then challenged with high doses of* Streptococcus pneumoniae*, mortality rates were increased above that of WT mice, correlating with increased proinflammatory cytokine production [11]. Yet, in another study in which* Plasmodium berghei* parasitemia was induced postimmunization, parasitemia was not impacted following intradermal immunization of Fc*γ*RIIB KO versus WT mice with a* Plasmodium berghei* vaccine [12]. Thus, while enhanced immunity and/or protection against infection was observed in naïve Fc*γ*RIIB KO versus WT mice, the absence of Fc*γ*RIIB had either negative or no impact on immunity and/or protection following immunization.

To further clarify the role of Fc*γ*RIIB in the generation of protective immunity against infection, we investigated the impact of Fc*γ*RIIB on* F. tularensis* (*Ft*) challenge before and after immunization with inactivated* Ft* (i*Ft*) in WT versus Fc*γ*RIIB KO mice.* Ft* is a gram-negative intracellular pathogen that in designated a Category A biothreat agent due to its extreme virulence [13–15]. Based on the published studies cited above using naïve Fc*γ*RIIB KO mice [9–11], we hypothesized that naïve mice challenged with* Ft* would be better protected in the absence of Fc*γ*RIIB. In contrast, based on the published studies above using immunized Fc*γ*RIIB KO mice [11, 12], we predicted a negative or very limited impact of the absence of Fc*γ*RIIB on survival following* Ft*-challenge of i*Ft*-immunized Fc*γ*RIIB KO versus WT mice. In fact, the opposite was observed in the case of both naïve and i*Ft*-immunized mice. Specifically, there was no difference in survival of naïve Fc*γ*RIIB KO versus WT mice challenged with* Ft*. In contrast, in the case of i*Ft*-immunized Fc*γ*RIIB KO versus WT mice, protection was significantly improved in Fc*γ*RIIB KO mice. The latter correlated with enhanced* Ft*-specific IgA production* in vivo*, enhanced recall responses in the form of increased IFN-*γ* production by* ex vivo* splenocytes incubated with i*Ft*, and reduced* in vivo* proinflammatory cytokine production 5 days after challenge. Thus, these studies demonstrate for the first time that the presence of Fc*γ*RIIB can significantly dampen immunity and protection against infection following immunization. These are also the first studies to suggest a role of Fc*γ*RIIB in the regulation of Ag-specific IgA production. Potential explanations for the above observations are discussed.

## 2. Materials and Methods

### 2.1. Reagents

RPMI medium was obtained from Cellgro Mediatech Inc. (Manassas, Va) into which was added 10% heat inactivated fetal bovine serum (FBS) (Hyclone Thermo Scientific, South Logan, Utah), 1% Pen/Strep (Hyclone Thermo Scientific, South Logan, Utah), 5 mL of 200 mM glutamine (100X) (Cellgro Mediatech Inc., Manassas, Va), 5 mL of 100 mM sodium pyruvate (100X) (Gibco Invitrogen, Grand Island, NY), 5 mL nonessential amino acids (AA) (100X) (Cellgro Mediatech Inc., Manassas, Va), and 250 *μ*L of 0.1 M 2-mercaptoethanol (2-ME) (Bio-rad, Hercules, CA). Media was then filter sterilized with a 0.22 *μ*m filter and stored at 4°C.* Ft*-live vaccine strain (LVS) was utilized in these studies and was cultured in Mueller Hinton Broth (MHB) medium consisting of 490 mL of distilled, deionized cell culture grade water (Cellgro Mediatech Inc., Manassas, Va), 10.5 g MHB (Becton Dickinson, Sparks, Maryland), 0.069 g anhydrous calcium chloride (Acros Organics, NJ), 0.105 g hydrous magnesium chloride (hexahydrate) (MP Biomedicals, Solon, OH), 5 mL of glucose (Sigma-Aldrich, St. Louis, Missouri), 5 mL of ferric pyrophosphate (Sigma-Aldrich, St. Louis, Missouri), and 10 mL isovitalex (Becton Dickinson, Sparks, Maryland). MHB media was adjusted to a pH of 6.8, filtered (0.22 *μ*m), and stored at 4°C for up to two weeks. Red blood cell (RBC) lysis buffer contained 4.13 g of ammonium chloride (Sigma-Aldrich, St. Louis, Missouri), 0.5 g of potassium bicarbonate (Sigma-Aldrich, St. Louis, Missouri), and 18.5 mg of EDTA (Sigma-Aldrich, St. Louis, Missouri) diluted in 500 mL of distilled water. The pH of the solution was adjusted to 7.2, after which the solution was filter-sterilized using a 0.22 *μ*m filter and stored at 4°C.

### 2.2. Splenocyte Isolation

Spleens were isolated from mice and passed through a 70 *μ*m cell strainer (Fisherbrand, Houston, TX). The single cell suspension was collected in a sterile petri dish containing approximately 3 to 5 mL of media. The cell suspension was washed and RBCs were lysed using a RBC lysis buffer. The cell suspension was then passed through a second 70 *μ*m cell strainer (Fisherbrand, Houston, TX) and collected in a 50 mL conical centrifuge tube containing approximately 10 mL of media. Cells were spun for 5 minutes at 1500 revolutions per minute (RPM) and resuspended in RBC lysis buffer. After 3 to 5 minutes (min), the reaction was quenched through the addition of 20 to 40 mL of media. Splenocytes were again spun for 5 min at 1500 RPM. Following resuspension, any residual tissue was removed via pipet or by again passing the splenocyte suspension through a 70 *μ*m filter (Fisherbrand, Houston, TX). Splenocytes were subsequently washed twice with fresh media twice and enumerated using trypan blue.

### 2.3. Mice

C57BL/6J mice were obtained from Jackson Laboratories (Bar Harbor, ME). Breeding pairs of Fc*γ*RIIB KO mice (B6; 129S4-Fcgr2b^tm1Ttk^/J) on a C57BL background were obtained from Jackson Laboratories and bred in the Animal Resources Facility at Albany Medical College. Mice of 6–15 weeks old were used in order to identify significant differences under conditions that include a relatively broad range of ages. All animals were housed and cared for according to guidelines approved by the Institutional Animal Care and Use Committee.

### 2.4. Generation of i*Ft*-Immunogen

i*Ft* was used as immunogen. Briefly,* Ft*-LVS organisms were grown in MHB medium (Becton Dickinson, Sparks, Maryland) at 37°C to a density of 0.5–1 × 10^9^ CFU/mL. 1 × 10^10^ CFU/mL live bacteria were then incubated in 1 mL of sterile PBS (Cellgro, Manassas, Virginia) containing 2% paraformaldehyde (Sigma-Aldrich, St. Louis, Missouri) for 2 hours at room temperature. The i*Ft*-organisms were then washed with sterile PBS three times. Inactivation was verified by culturing a 100 *μ*L sample (~1 × 10^9^ i*Ft*-organisms) on chocolate agar plates (Becton, Sparks, Maryland) for 7 to 10 days. The i*Ft* preparations were stored at −20°C in PBS.

### 2.5. Immunization and Challenge Studies

Mice were divided into groups consisting of 6–8 mice per group. Each mouse was anesthetized by intraperitoneal (i.p.) injection of 20% ketamine (Vedco, St. Joseph, Missouri) plus 5% xylazine (Lloyd, Shenandoah, Iowa) diluted in sterile cell culture grade water (Cellgro, Manassas, Virginia). Mice were subsequently administered i.n. either 20 *μ*L of PBS (vehicle) or 2 × 10^7^ i*Ft*-organisms in 20 *μ*L of PBS. Unless otherwise indicated, mice were immunized on day 0 and boosted on day 21. Immunized mice were then challenged on day 35 i.n. with either 4 × LD_50_ or 16 × LD_50_ of live* Ft*-LVS and subsequently monitored for survival for a minimum of 21 days.

### 2.6. Bacterial Burden Quantitation

Mice were immunized and subsequently challenged with a sublethal dose (approximately 0.4 × LD_50_) of live* Ft*-LVS. Five days after challenge, mice were sacrificed and tissues were collected. Tissues were homogenized with a mechanical bead beater and serially diluted with sterile PBS. Diluted tissue samples were plated on chocolate agar and CFU colonies were enumerated approximately 72 hours later.

### 2.7. Antibody Quantitation

Total mouse IgM, IgG, and IgA were measured by ELISA. Kits were purchased (Immunology Consultants Laboratory, Portland, OR) and the manufacturers protocol was followed. All reagents were brought to room temperature. Samples and standards were serially diluted and 100 *μ*L/well were added to plates precoated with anti-IgM, anti-IgG, or anti-IgA Abs. Plates were incubated at room temperature for 60 minutes. Following the incubation, plates were aspirated and washed, and 100 *μ*L of the appropriate enzyme-Ab conjugate was added to each well. After 30 minutes, the plates were again washed and substrate solution was added. The reaction was stopped after 10 minutes with stop solution and the plates were read at 450 nm using a microplate reader (VersaMax, Molecular Devices, Sunnyvale, California).* Ft*-specific Ab production was also measured by ELISA. ELISA plates (Corning, Corning, New York) were coated with 50 *μ*L/well of live* Ft*-LVS (5 × 10^7^ CFU/mL in carbonate buffer containing 2.15 g sodium bicarbonate and 2.62 g sodium carbonate (Sigma-Aldrich, St. Louis, Missouri) diluted in 500 mL of sterile cell culture grade water (Cellgro, Manassas, Virginia) at pH 9.6–9.8), overnight at 4°C. The plates were then washed three times with 200 *μ*L/well of PBS containing 0.5% bovine serum albumin (BSA) (Baxter Healthcare, Deerfield, Illinois) and 0.002% sodium azide (Sigma-Aldrich, St. Louis, Missouri). Plates were then blocked at 4°C for 2 hours with 200 *μ*L/well of PBS containing 5% BSA and 0.02% sodium azide. Plates were again washed and serial dilutions of sera or bronchoalveolar lavage fluids (BALF) were added to the plates (50 *μ*L/well) and incubated for 2 hours at 4°C. After washing, alkaline phosphatase conjugated anti-mouse Ab specific for total mouse Ab (Sigma-Aldrich, St. Louis, Missouri), IgG (Sigma-Aldrich, St. Louis, Missouri), IgA (Sigma-Aldrich, St. Louis, Missouri), IgG1 (Southern Biotech, Birmingham, AL), IgG2c (Abcam, Cambridge, MA), or IgM (Sigma-Aldrich, St. Louis, Missouri) were added and incubated for 1 hour at 4°C. Plates were washed and then 100 *μ*L/well of alkaline phosphatase substrate (Sigma, St. Louis, MO) was added. All samples were read at 405 nm using a microplate reader (VersaMax, Molecular Devices, Sunnyvale, California) following a 5-second (sec) shake.

### 2.8. Adoptive Transfer Studies

Mice were immunized with i*Ft* i.n. as described above and on day 35 mice were anesthetized and blood was collected via percutaneous cardiac puncture. Clotted blood samples were then spun at 8000 RPM for 10 minutes and serum was harvested and pooled. Following heat (complement) inactivation at 55°C for 30 minutes, serum was spun at 4000 RPM for 10 minutes, aliquoted, and frozen at −20°C until use. For adoptive transfer experiments, mice were administered i.p. 250 *μ*L of PBS (vehicle), serum from PBS immunized mice, or serum i*Ft*-immunized mice. Mice were then anesthetized and challenged with the indicated dose of* Ft*-LVS 24 hours after adoptive transfer. Survival was monitored for 21 days.

### 2.9. *Ex Vivo* Splenocyte Activation (Recall Response) Assay

On day 35 after primary immunization, spleen cells were harvested and diluted in medium to a concentration of 5 × 10^6^ spleen cells/mL. 1000 *μ*L of cells was added to wells of a 24-well plate (Costar Corning, Corning, NY) plate, each well containing an equivalent number of i*Ft*-organisms. Alternatively 100 *μ*L of cells was added to wells of a U-bottom 96-well plate (Falcon BD, Franklin Lakes, NJ) that contained a concentration range between 2.5 and 20 i*Ft* per splenocyte. Plates were incubated at 37°C for up to 7 days in a humidity chamber to prevent medium evaporation. Supernatants were collected at 1, 3, 5, and 7 days and frozen at −20°C until they were analyzed. Samples were analyzed for cytokines via cytometric bead array (CBA) multiplex assay (BD Biosciences-BD Pharmingen, Sparks, Maryland). Data was acquired on a FACSArray Instrument and analyzed using CBA software version 1.0.1 (BD Immunocytometry Systems, Sparks, Maryland).

### 2.10. Cytokine Quantitation in Lungs and BALF

WT and Fc*γ*RIIB KO mice were immunized i.n. and on day 35 mice were given a sublethal challenge i.n. with approximately 0.4 × LD_50_ of* Ft*-LVS. Tissues were collected five days after challenge. Tissues were homogenized and centrifuged, and supernatants were collected. Luminex assay was performed on tissue supernatants to determine* in vivo* cytokine levels and to assess inflammation. The Luminex assay is a multiplex system for quantitation and detection of multiple cytokines in a single sample.

### 2.11. Statistical Analysis

The method of statistical analysis for each figure is described in the respective figure legends.

## 3. Results

### 3.1. Naïve Fc*γ*RIIB KO Mice Exhibit Increased Levels of Total Serum IgG versus That of Naïve WT Mice

Given the key role Ab plays in initiating Fc*γ*RIIB-mediated downmodulation of the immune response, we sought to verify prior studies demonstrating increased levels of total serum IgG in Fc*γ*RIIB KO versus WT mice [16]. We also examined serum levels of total IgM and total IgA, as well as* Ft*-specific IgM and IgG, since mice have propensity for low basal levels of natural* Ft*-specific Ab [17]. As shown in Table 1, we observed no difference in total IgM levels between WT and Fc*γ*RIIB KO mice. However, naïve Fc*γ*RIIB KO mice exhibited higher levels of total serum IgG than their WT counterparts. Total IgA also appeared somewhat elevated in Fc*γ*RIIB KO versus WT mice. In regard to the presence of natural* Ft*-specific IgM and IgG Ab, there were no significant differences between Fc*γ*RIIB KO versus WT mice, although the median titer for* Ft*-specific IgG was higher in Fc*γ*RIIB KO mice (Table 1).

### 3.2. There Is No Difference in Survival of Naive Fc*γ*RIIB KO versus Naïve WT Mice Challenged with* Ft*-LVS

**Figure 1:**
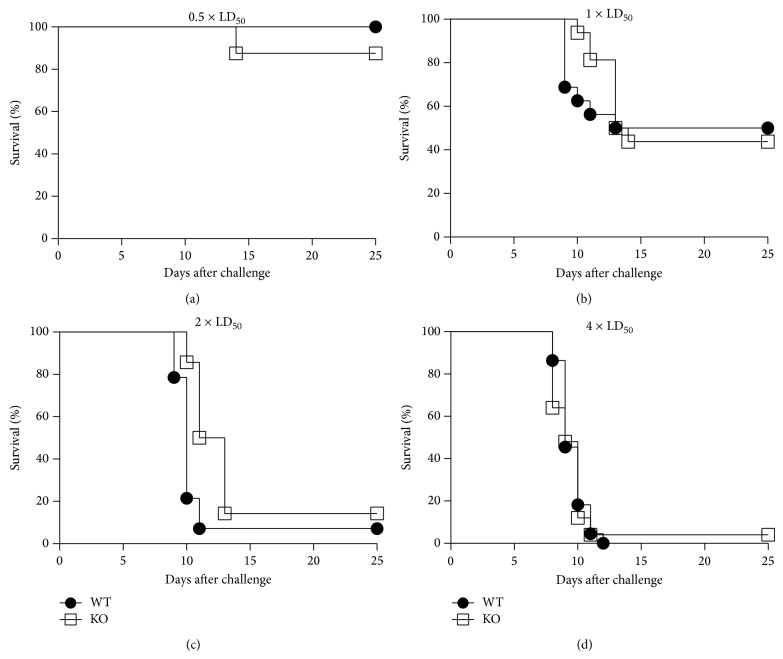
There is no difference in survival of naive Fc*γ*RIIB KO versus naïve WT mice challenged with* Ft*-LVS. Naïve WT C57BL/6J and Fc*γ*RIIB KO mice were challenged i.n. with 0.5 × LD_50_ (a), 1 × LD_50_ (b), 2 × LD_50_ (c), or 4 × LD_50_ (d) of* Ft*-LVS and survival was monitored for 25 days after challenge. Panel (a) represents 8 mice/group while panels (b)–(d) represent between 14 and 25 mice/group. Statistical analysis was determined by a contingency table analysis and two-tailed Fisher's exact test on survival at day 25.

Based on published studies demonstrating a beneficial effect on the immunity and/or protection of naïve Fc*γ*RIIB KO versus WT mice following infectious disease challenge [10, 11], we expected that the absence of Fc*γ*RIIB would enhance survival of naïve Fc*γ*RIIB KO mice challenged with* Ft*-LVS. In fact, we observed no significant difference in survival between naïve Fc*γ*RIIB KO and WT mice at all challenge doses tested (Figures 1(a)–1(d)). Thus, the elevated median response levels of natural* Ft*-specific serum IgG Ab in naïve Fc*γ*RIIB KO mice versus WT mice (Table 1) had no impact on survival (Figures 1(a)–1(d)).

### 3.3. *Ft*-Specific IgA, but Not IgG, Is Significantly Increased in Fc*γ*RIIB KO versus WT Mice following i*Ft*-Immunization

**Figure 2:**
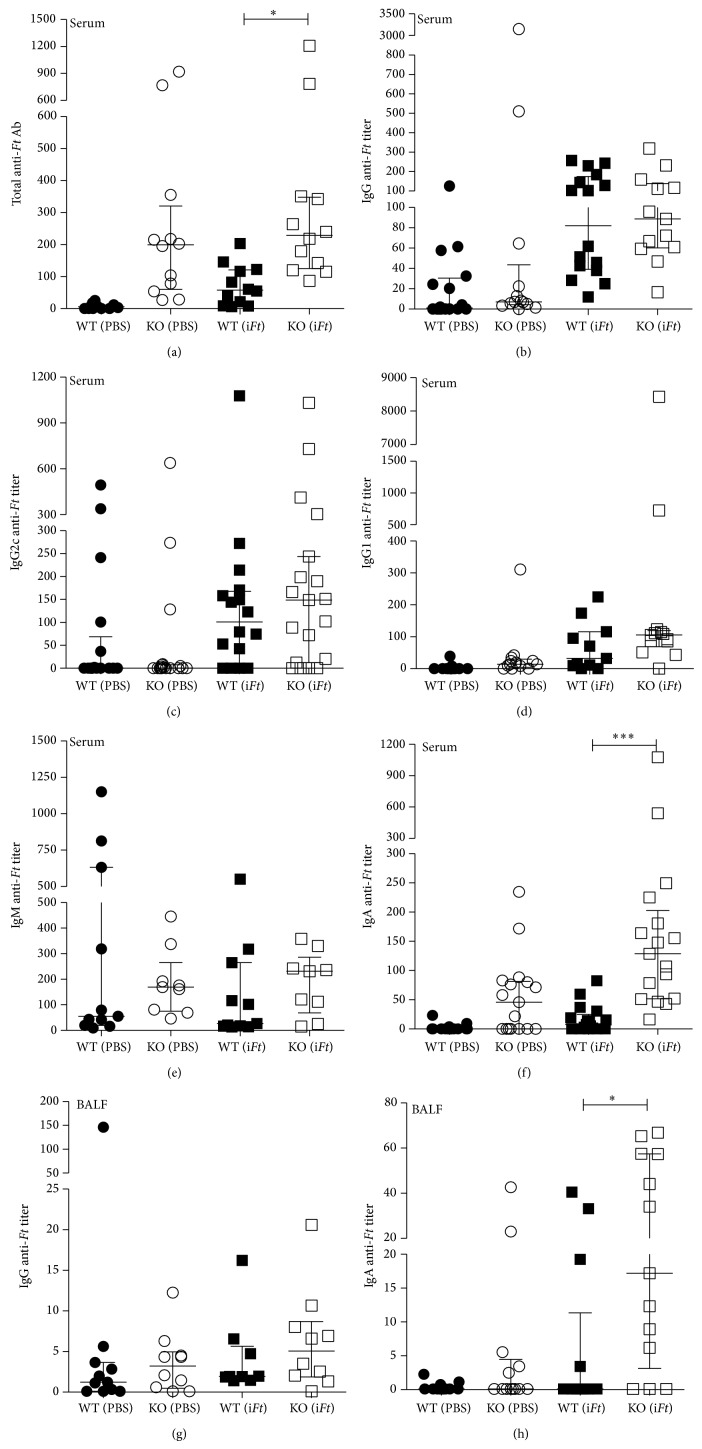
*Ft*-specific IgA, but not IgG, is significantly increased in Fc*γ*RIIB KO versus WT mice following i*Ft*-immunization. Fc*γ*RIIB KO and WT mice were immunized i.n. with 20 *μ*L of PBS (vehicle) or 2 × 10^7^ i*Ft-*organisms in 20 *μ*L of PBS on day 0 and boosted on day 21. Serum was then collected on day 35 and levels of total* Ft*-specific Ab (a),* Ft*-specific IgG (b),* Ft*-specific IgG2c (c),* Ft*-specific IgG1 (d),* Ft*-specific IgM (e), and* Ft*-specific IgA (f) were determined via ELISA. Titers of* Ft*-specific IgG (g) and* Ft*-specific IgA (h) in BALF were similarly determined. Each symbol represents a single mouse. The 50% maximum of the highest experimental OD was determined and all experimental OD values were transformed to their respective log values. A log (agonist) versus response-variable slope (four parameters) nonlinear regression (curve fit) was performed on the log values, and the unknowns were interpolated. The inverse log was taken on the interpolated unknowns to determine the final titers for each individual mouse sample (error bars represent median with interquartile range of data distribution). Statistics were performed by adding 0.1 to the determined titer value and taking the log of this new value to correct for heteroscedasticity. A one-way ANOVA along with a Tukey's multiple comparison test was performed, and median and interquartile range is graphed. ^∗∗∗^ *P* < 0.001; ^∗^ *P* < 0.05.

A number of published studies have demonstrated that in the absence of Fc*γ*RIIB, Ag-specific IgG production is enhanced upon immune stimulation [18–20]. Thus, we examined the production of* Ft*-specific Ab, including IgG, IgA, and IgM, in the serum and BALF of Fc*γ*RIIB KO versus WT mice immunized with i*Ft*. In some cases, the titers obtained were highly variable between individual mice, which likely reflects not only normal mouse-to-mouse variation but also differences in immune responsiveness due to differences in the age of the individual mice and its effects on B cell repertoires [21]. Despite the latter, we did observe a significant increase in the production of total* Ft*-specific serum Ab in Fc*γ*RIIB KO versus WT mice following i*Ft*-immunizations (Figure 2(a)). While there were no significant differences in the levels of* Ft*-specific serum IgG, IgG2c, IgG1, or IgM in the serum of these mice (Figures 2(b), 2(c), 2(d), or 2(e), resp.),* Ft-*specific IgA levels in serum were significantly increased (Figure 2(f)). While there is not a dramatic increase in the median response in total anti-*Ft* Ab titer between PBS and i*Ft*-immunized Fc*γ*RIIB KO mice (Figure 2(a)), there is a significant increase in the median levels of IgG2c in i*Ft*-immunized Fc*γ*RIIB KO mice versus PBS-immunized Fc*γ*RIIB KO mice (*P* < 0.01) (Figure 2(c)). This suggests that despite the former observation regarding total anti-*Ft* Ab, i*Ft*-immunized Fc*γ*RIIB KO mice produce increased levels of potentially high affinity IgG2c anti-*Ft* Ab. Lastly, there were no significant differences in the levels of* Ft*-specific IgG in the BALF of these mice, while the levels of* Ft-*specific IgA in the BALF of i*Ft*-immunized Fc*γ*RIIB KO mice were significantly higher than that of WT mice (Figure 2(h)). The presence of* Ft*-specific Abs in naïve (Table 1) or unimmunized mice has been previously observed in mouse [17] and humans [22, 23] and is likely due to the presence of B1 cells producing natural antibodies stimulated in response to normal flora or self-Ag [24, 25].

### 3.4. i*Ft*-Immunized Fc*γ*RIIB KO Mice Exhibit Increased Protection against a Lethal* Ft*-LVS Challenge as Compared to Their WT Counterparts

**Figure 3:**
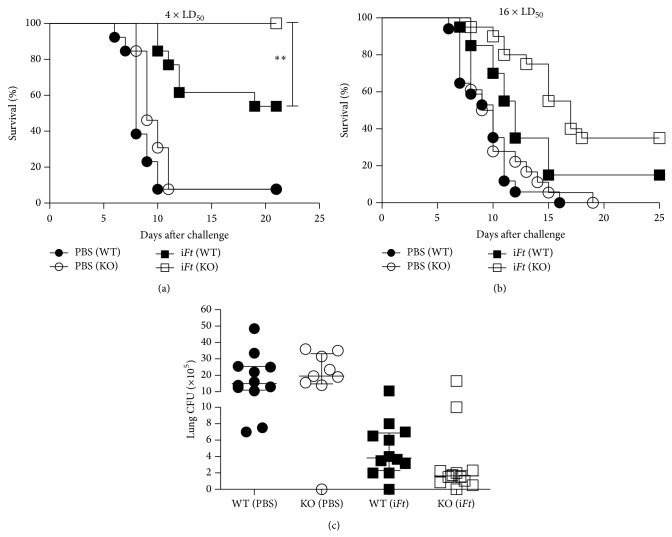
i*Ft*-immunized Fc*γ*RIIB KO mice exhibit increased protection against a lethal* Ft*-LVS challenge as compared to their WT counterparts. WT C57BL/6J and Fc*γ*RIIB KO mice were immunized i.n. with 20 *μ*L of PBS (vehicle) or 2 × 10^7^ i*Ft*-organisms in 20 *μ*L of PBS on day 0 and boosted on day 21. On day 35 mice were challenged i.n. with approximately 4 × LD_50_ (a) or 16 × LD_50_ (b) of* Ft*-LVS. Survival was then monitored for 21 to 25 days. Panel (a) represents between 12 and 14 mice/group while panel (b) represents between 17 and 20 mice/group. Statistical analysis was determined by a contingency table analysis and two-tailed Fisher's exact test on survival at day 21. ^∗∗^ *P* < 0.01. In addition, bacterial burden in the lungs of challenged mice was also determined. WT C57BL/6J and Fc*γ*RIIB KO mice were immunized i.n. with 20 *μ*L of PBS (vehicle) or 2 × 10^7^ i*Ft*-organisms in 20 *μ*L of PBS on day 0 and boosted on day 21. On day 35 mice were given a sublethal challenge i.n. with approximately 0.4 × LD_50_ of* Ft*-LVS. Five days after challenge lungs were collected, homogenized, and plated on chocolate agar to determine bacterial burden as described in Section 2. Each symbol represents a single mouse. Statistical analysis of the tissue bacterial burden was done via a nonparametric one-way ANOVA (Kruskal-Wallis test) coupled with a Dunn's multiple comparison after test. While there appeared to be a substantial reduction in bacterial burden in the majority of i*Ft*-immunized Fc*γ*RIIB KO versus WT mice, the difference was not significant based on this analysis.

Given the integral role Ag-specific IgG plays in Fc*γ*RIIB-mediated immune modulation and the absence of increased levels of* Ft*-specific IgG in i*Ft*-immunized Fc*γ*RIIB KO versus WT mice (Figure 2), we sought to determine whether there would be any impact of the presence versus absence of Fc*γ*RIIB on protection of i*Ft*-immunized mice. Despite the lack of a significant increased* Ft*-specific IgG in Fc*γ*RIIB KO versus WT mice and in contrast to studies using naïve mice (Figure 1), at a challenge dose of 4 × LD_50_, the survival of i*Ft*-immunized Fc*γ*RIIB KO mice was significantly better than that of WT mice (100% versus 50% resp.) (Figure 3(a)). Similar to that observed in naïve mice (Figure 1), there was no significant difference in survival between Fc*γ*RIIB KO and WT mice immunized with PBS (Figure 3(a)). When the challenge dose was increased to 16 × LD_50_, the overall level of survival decreased. However, a slight increase in the survival of i*Ft*-immunized Fc*γ*RIIB KO versus WT mice was still apparent (Figure 3(b)). The increased survival also correlated with a reduction in the median bacterial burden in i*Ft*-immunized Fc*γ*RIIB KO (Figure 3(c)).

### 3.5. Adoptive Transfer of Serum from i*Ft*-Immunized Fc*γ*RIIB KO and WT Mice to Naïve Fc*γ*RIIB KO and WT Mice, Respectively, Does Not Replicate the Differences in Survival Observed in i*Ft*-Immunized Fc*γ*RIIB KO versus WT Mice

We surmised that while there were no differences in* Ft*-specific IgG levels in i*Ft*-immunized Fc*γ*RIIB KO versus WT mice (Figure 2), the observed increases in* Ft*-specific IgA in the serum and BALF of Fc*γ*RIIB KO versus WT mice might be responsible for the increased protection observed (Figure 3). To test this, we adoptively transferred serum from i*Ft*-immunized Fc*γ*RIIB KO and WT mice into their naive counterparts, in an effort to recapitulate the difference in survival obtained in Figure 3. As demonstrated previously by others [26], adoptive transfer of serum to naïve mice did increase protection against* Ft*-LVS challenge. However, a significant increase in protection in naïve Fc*γ*RIIB KO mice receiving serum from i*Ft*-immunized Fc*γ*RIIB KO mice versus that of WT mice was not observed (Table 2).

### 3.6. Splenocytes from i*Ft*-Immunized Fc*γ*RIIB KO versus WT Mice Exhibit Enhanced IFN-*γ*, IL-10, and TNF-*α* Production in Response to i*Ft* Added* Ex Vivo*

**Figure 4:**
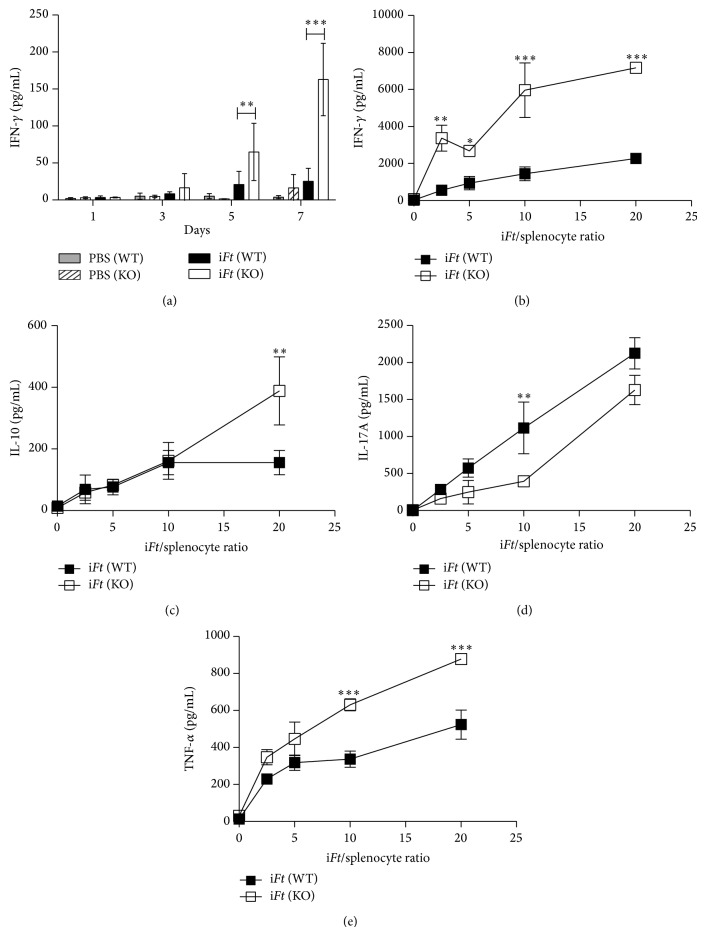
Splenocytes from i*Ft*-immunized Fc*γ*RIIB KO versus WT mice exhibit enhanced IFN-*γ*, IL-10, and TNF-*α* production in response to i*Ft* added* ex vivo*. WT C57BL/6J and Fc*γ*RIIB KO mice were immunized i.n. with 20 *μ*L of PBS (vehicle) or 2 × 10^7^ i*Ft*-organisms in 20 *μ*L of PBS on day 0 and boosted on day 21. (a) Splenocytes were then harvested from individual mice on day 35 and stimulated at a ratio of one i*Ft* per splenocyte. Supernatants were then harvested at the indicated days. (b) Fc*γ*RIIB KO and WT mice were immunized and splenocytes were harvested as previously described. Splenocytes were then stimulated at increasing i*Ft* to splenocyte ratios. Supernatants were harvested five days later and analyzed for IFN-*γ*, (c) IL-10, (d) IL-17A, and (e) TNF-*α* via CBA assay. A two-way ANOVA and a Bonferroni posttest was performed on the CBA determined sample concentrations to determine statistical significance, and mean and standard deviation is graphed. ^∗∗∗^ *P* < 0.001; ^∗∗^ *P* < 0.01; ^∗^ *P* < 0.05.

Given that IFN-*γ*, a Th1 cytokine, has been shown to play an important role in protection against* Ft*-infection [13, 27–30] and that Fc*γ*RIIB has been shown to downmodulate cellular immune responses, including Th1 responses [9, 11], we examined the impact of Fc*γ*RIIB's absence on the production of IFN-*γ* by splenocytes from i*Ft*-immunized Fc*γ*RIIB KO versus WT mice restimulated* ex vivo* with i*Ft*. Consistent with the increased protection observed in i*Ft*-immunized Fc*γ*RIIB KO versus WT mice (Figure 3) and a critical role for IFN-*γ* in mediating protection against* Ft*-infection, within three days following restimulation with i*Ft*, splenocytes from i*Ft*-immunized Fc*γ*RIIB KO mice exhibited increased levels of IFN-*γ* compared to that of WT splenocytes (Figure 4(a)). This remained the case at a series of i*Ft*/splenocyte ratios ranging between 2.5 and 20 : 1 (Figure 4(b)). Furthermore, in this same experiment, we also examined the production of IL-10 and IL-17, since IL-10 restrains IL-17-induced lung pathology following pulmonary* Ft*-LVS infection [31]. We demonstrate that i*Ft*-immunized Fc*γ*RIIB KO mice produce increased levels of IL-10 and lower levels of IL-17A than i*Ft*-immunized WT mice, consistent with the ability of IL-10 to limit IL-17 induced pathology in Fc*γ*RIIB KO mice (Figures 4(c) and 4(d)), leading to increased survival (Figure 3). Finally, we demonstrate that i*Ft*-immunized Fc*γ*RIIB KO mice produce higher levels of TNF-*α* compared to i*Ft*-immunized WT mice (Figure 4(e)), another cytokine that also plays an essential role in survival against a primary* Ft*-infection [32].

### 3.7. Levels of Proinflammatory Cytokines in the Lungs of i*Ft*-Immunized Fc*γ*RIIB KO versus WT Mice Were Reduced 5 Days after Challenge

**Figure 5:**
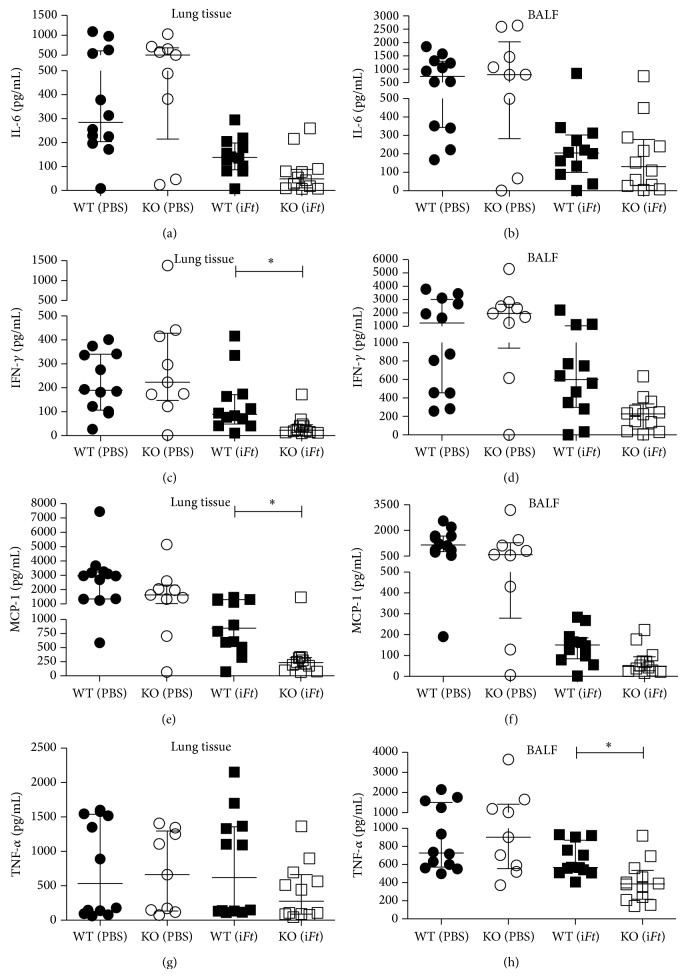
Levels of proinflammatory cytokines in the lungs of i*Ft*-immunized Fc*γ*RIIB KO versus WT mice were reduced 5 days after challenge. WT C57BL/6J and Fc*γ*RIIB KO mice were immunized i.n. with 20 *μ*L of PBS (vehicle) or 2 × 10^7^ i*Ft*-organisms in 20 *μ*L of PBS on day 0 and boosted on day 21. On day 35 mice were given a sublethal challenge i.n. with approximately 0.4 × LD_50_. Five days after challenge lung tissue was collected, homogenized, and centrifuged and supernatants were collected. Luminex assay was performed on tissue supernatants to determine* in vivo* cytokine levels. Each symbol represents a single mouse. A value of 0.1 was added to the average cytokine concentration to correct for heteroscedasticity. A one-way ANOVA along with a Tukey's multiple comparison test was performed on the log of the average cytokine concentration of lung and BALF samples from individual mice to determine statistical significance, and median and interquartile range is graphed. ^∗^ *P* < 0.05.

We have previously observed that although immunization with i*Ft* initially stimulates increased production of inflammatory cytokines, including IFN-*γ*, on days 1–3, an overall reduction in such cytokines occurs* in vivo* between days 5 and 7 after challenge. Furthermore, the latter decrease correlates with increased protection [13, 33]. Therefore, we also analyzed inflammatory cytokine levels in the lungs of i*Ft*-immunized Fc*γ*RIIB KO versus WT 5-day mice after challenge. Consistent with our prior observations, significantly lower levels of IFN-*γ* and MCP-1 in the lungs and TNF-*α* in the BALF of i*Ft*-immunized Fc*γ*RIIB KO versus WT mice were observed (Figures 5(c), 5(e), and 5(h), resp.). There were also reductions in the median cytokine levels for IL-6 and TNF-*α* in the lungs of these same mice (Figures 5(a) and 5(g), resp.), as well as IL-6 (Figure 5(b)), IFN-*γ* (Figure 5(d)), and MCP-1 (Figure 5(f)) in BALF.

## 4. Discussion

### 4.1. The Impact of Fc*γ*RIIB on Primary Infection

A very limited number of studies thus far have focused on the role of Fc*γ*RIIB in resolving primary infections [9–12]. Specifically, they suggest that, during primary infection, the absence of Fc*γ*RIIB results in an improved immune response to infection, which can be beneficial to the host [9–11]. In contrast, other studies suggest that, following immunization, the absence of Fc*γ*RIIB leads to an overproduction of cytokines and potential septic shock [11] or no differences in immunity and/or protection [11, 12]. However, our studies using the* Ft*-infectious disease model demonstrate that the absence of Fc*γ*RIIB has little or no impact on the outcome of survival following primary infection, while its absence following vaccination and challenge increases the protective efficacy of the vaccine. A number of likely explanations exist for our observations. In regard to primary infection of naïve mice, our studies and those of others have demonstrated Fc*γ*RIIB deficient mice exhibit higher levels of total IgG [6, 16]. Our studies also demonstrate that there is no significant difference in* Ft*-specific IgG or total IgA Abs in naïve Fc*γ*RIIB KO versus WT mice (Table 1). The latter, in addition to the fact that the formation of* Ft*-anti-*Ft* Ab complexes would be necessary to actively engage Fc*γ*RIIB and impact immunity and protection, may explain a failure to see differences in protection between naïve Fc*γ*RIIB KO and WT mice. Furthermore, the generation of* Ft*-specific IgG in response to infection would not be expected to occur until approximately day 7 after infection [26], the same point at which mice generally succumb to infection (Figure 1).

### 4.2. The Impact of Fc*γ*RIIB on Infection following Vaccination

In contrast to studies involving immunization against* S. pneumoniae* and subsequent challenge [11], i*Ft*-immunized mice are better protected when Fc*γ*RIIB is absent (Figure 3). This is also despite the lack of significant differences in the levels of* Ft*-specific IgG production in Fc*γ*RIIB KO versus WT mice following i*Ft*-immunization (Figure 2). Importantly, however, both IgG and IgA can mediate protection against* Ft*-infection [13, 29, 30, 34–38]. Thus, the significant increase in* Ft*-specific IgA production in i*Ft*-immunized Fc*γ*RIIB KO versus WT mice (Figure 2) could explain the increased protection observed with i*Ft*-immunized Fc*γ*RIIB KO versus WT mice. Never the less, a role for* Ft*-specific IgA in mediating the increased protection observed (Figure 3) is not supported by adoptive transfer studies. Specifically, no significant difference in protection was observed in naïve recipient Fc*γ*RIIB KO versus WT mice following adoptive transfer of sera from i*Ft*-immunized Fc*γ*RIIB KO or WT donors, respectively (Table 2). However, cellular immune responses can also play a crucial role in survival against a lethal* Ft*-challenge [13, 27–30, 34–38]. In this regard, we do show splenocytes from i*Ft*-immunized Fc*γ*RIIB KO mice incubated with i*Ft ex vivo* produce substantially more IFN-*γ*, IL-10, and TNF-*α* than their WT counterparts, while producing less IL-17, which has been associated with increased pathology (Figure 4) [31]. Thus, this observation more likely explains the enhanced protection observed in i*Ft*-immunized Fc*γ*RIIB KO mice. However, a role for* Ft*-specific IgA cannot be totally excluded, in that our prior studies have demonstrated the requirement for both IgA and IFN-*γ* in protection of i*Ft*-immunized mice following* Ft*-LVS challenge [13, 33]. Lastly, our studies, and those of others, have demonstrated that a reduction in the levels of proinflammatory cytokines 5–7 days after challenge, correlates with increased protection in the* Ft*-infectious disease model [13, 29, 33, 37, 39]. Accordingly, we also observed a significant reduction in proinflammatory cytokines in the lungs of i*Ft*-immunized Fc*γ*RIIB KO versus WT mice 5 days after challenge (Figure 5). Furthermore, this decrease in proinflammatory cytokines in lungs of i*Ft*-immunized Fc*γ*RIIB KO versus WT mice at 5 days after challenge also correlated with a decrease in bacterial burden in the lungs of these animals, reflecting the ability of i*Ft*-immunized Fc*γ*RIIB KO mice to better control* Ft*-infection than their WT counterparts (Figure 3).

### 4.3. Consideration of Fc*γ*RIIB's Regulatory Role in the Development of Vaccines

We have recently demonstrated that targeting i*Ft* to Fc receptor (FcR) in the form of monoclonal Ab- (mAb-) i*Ft* complex administered i.n., provides superior protection to that of i*Ft* [13]. However, a major concern regarding Fc*γ*R-targeted vaccines, when utilizing Fc to target vaccine Ags, is their ability to bind to Fc*γ*RIIB, as well as activating Fc*γ*Rs, which could potentially dampen the response to vaccination and thereby limit efficacy. Thus, FcR-targeted vaccines that bypass Fc*γ*RIIB in favor of activating Fc*γ*R, such as one recently developed in our laboratory, which targets the activating Fc*γ*R (human Fc*γ*RI) [40], could significantly increase the efficacy of such vaccines. Alternatively, as we have demonstrated in this paper, Fc*γ*RIIB can also limit the stimulatory capacity of non-FcR-targeted vaccines. Thus, the use of Fc*γ*RIIB antagonists as vaccine adjuvants could significantly enhance the efficacy of vaccines in general.

## 5. Conclusions

This study is the first to demonstrate that (1) the absence of Fc*γ*RIIB does not affect the susceptibility of mice to a primary infection with the intracellular Category A mucosal pathogen* F. tularensis*; (2) that in the absence of Fc*γ*RIIB, both humoral and cellular immunity are enhanced following immunization with the inactivated vaccine i*Ft*; (3) that the level of Ag-specific IgA produced in response to vaccination can be impacted by the presence/absence of Fc*γ*RIIB; (4) that the enhanced immune responses observed (*Ft*-specific IgA and IFN-*γ* production) following i*Ft*-immunization correlate with increased protection of Fc*γ*RIIB KO versus WT mice after lethal mucosal challenge with* Ft* LVS. Thus, these studies further expand our knowledge regarding the role of Fc*γ*RIIB plays in the immune response to infection, while also providing further impetus for developing vaccines geared toward modulating the inhibitory activities of this receptor.

## Figures and Tables

**Table 1:** Relative differences in Ab levels between naïve WT and naïve Fc*γ*RIIB KO mice.

Experimental group	Response measured	WT mice (median response)	KO mice (median response)	Fold change^c^
Naïve before challenge	Total serum IgM^a^	451	441	1.0
	Total serum IgG^a^	758	1134	1.5^*^
	Total serum IgA^a^	14	20	1.4
	*Ft*-specific serum IgM^b^	88	85	1.0
	*Ft*-specific serum IgG^b^	33	92	2.8

^a^ *μ*g/mL of Ab.

^b^Ab titer.

^c^Fold change was caculated by deviding KO mice (median response) by WT mice (median response).

^*^ *P* < 0.05.

**Table 2:** Summary of adoptive transfer survival studies.

Group	Donor	Immunogen	Recipient	Percent protection	Mouse number	Statistical comparison	Significance (*P* value^a^)
A	WT	PBS	WT	6.67	*n* = 15		
B	KO	PBS	KO	26.67	*n* = 15	A versus B	*P* = 0.3295
C	WT	i*Ft *	WT	53.33	*n* = 15		
D	KO	i*Ft *	KO	62.5	*n* = 16	C versus D	*P* = 0.7224

^a^The *P* values were determined by performing a contingency table analysis and two-tailed Fisher's exact test on survival at day 21 after challenge.
